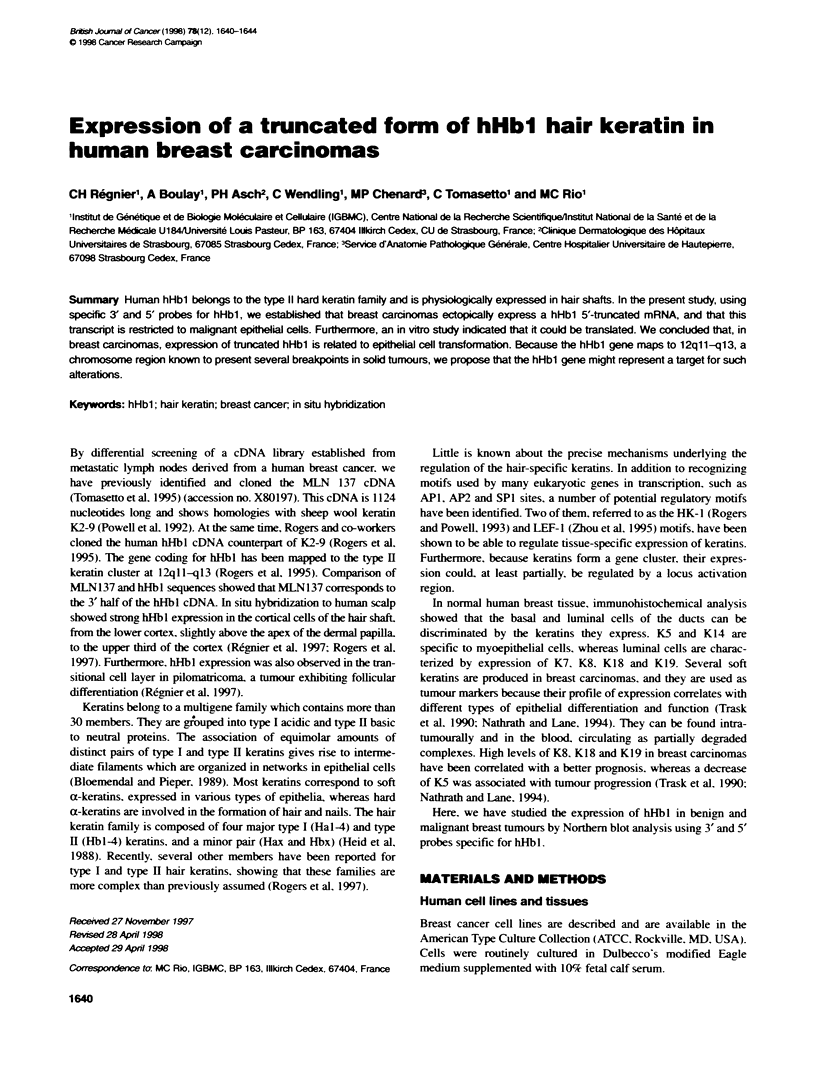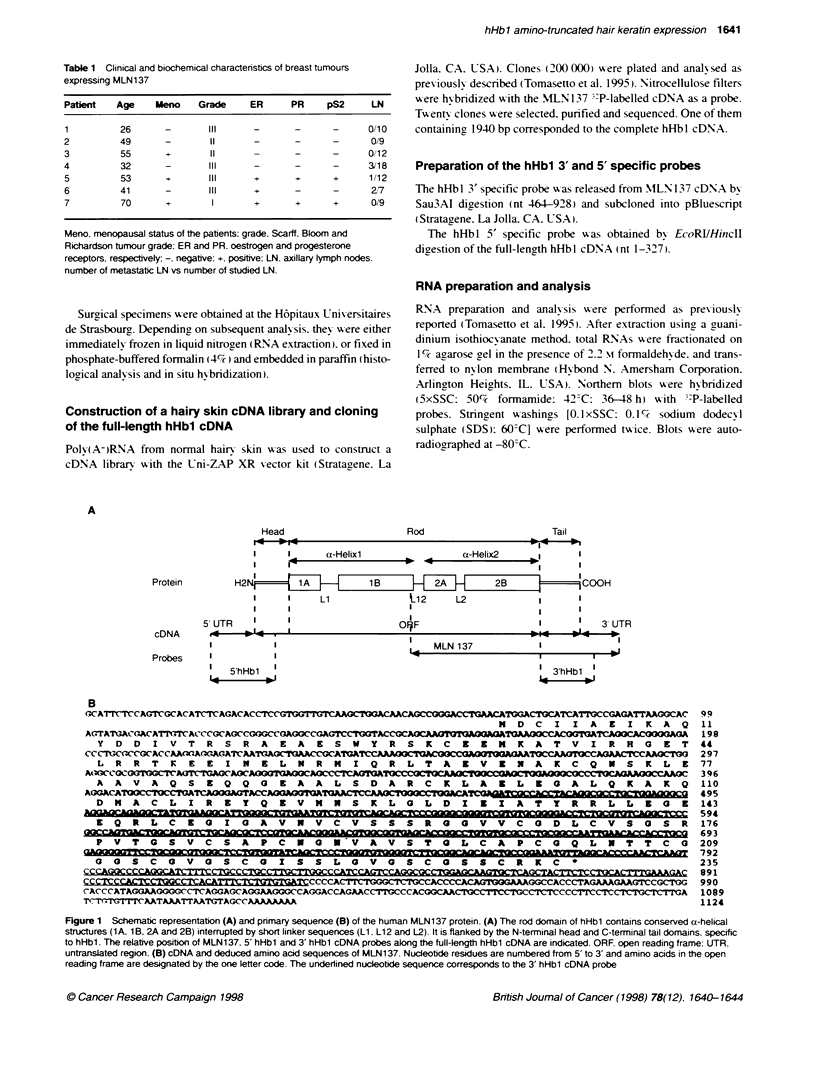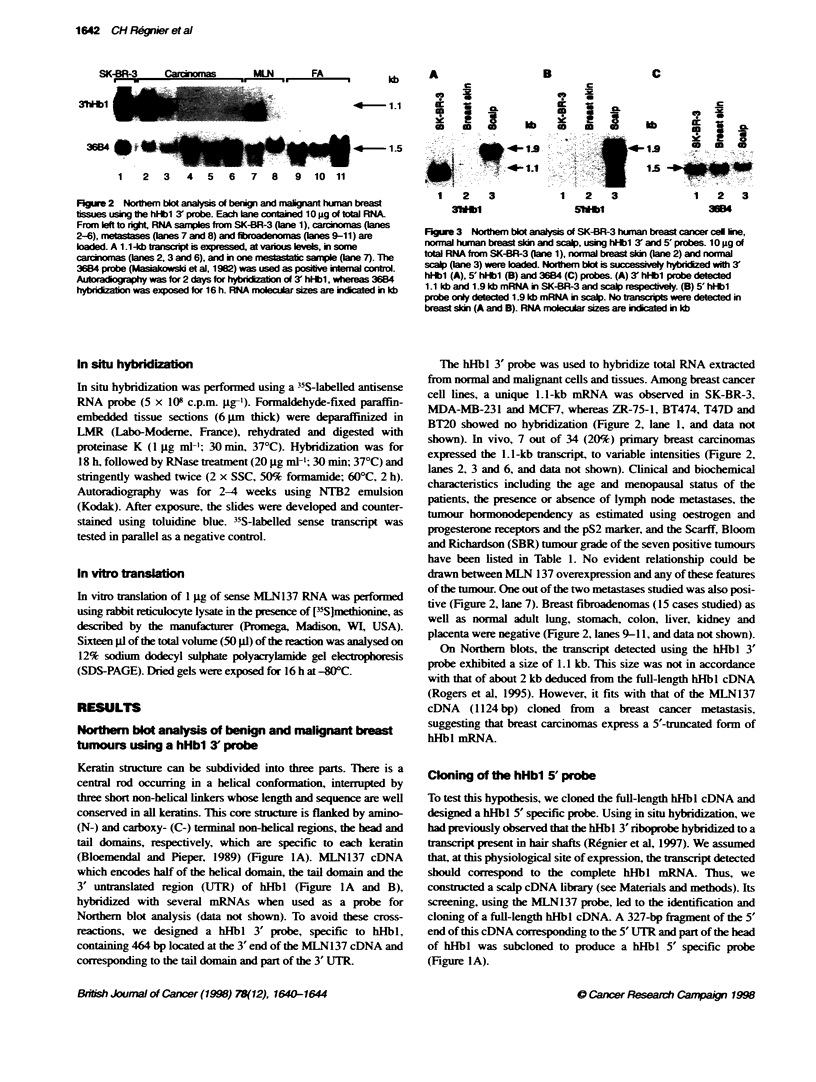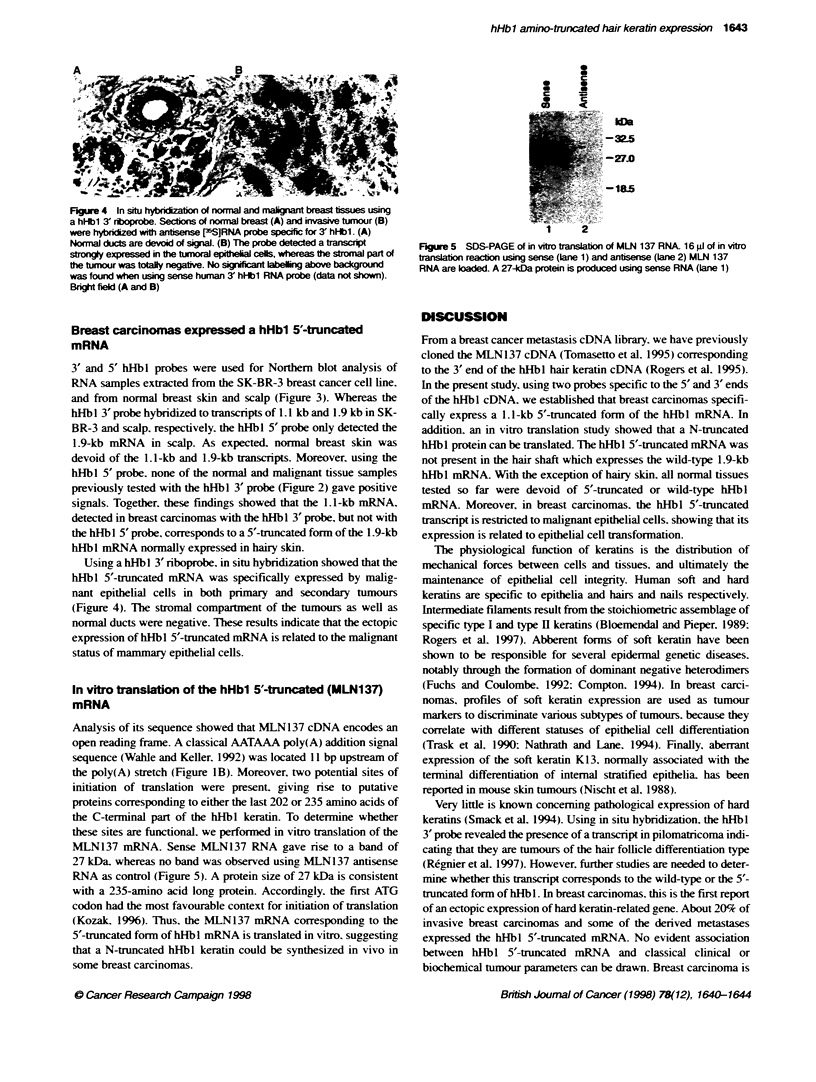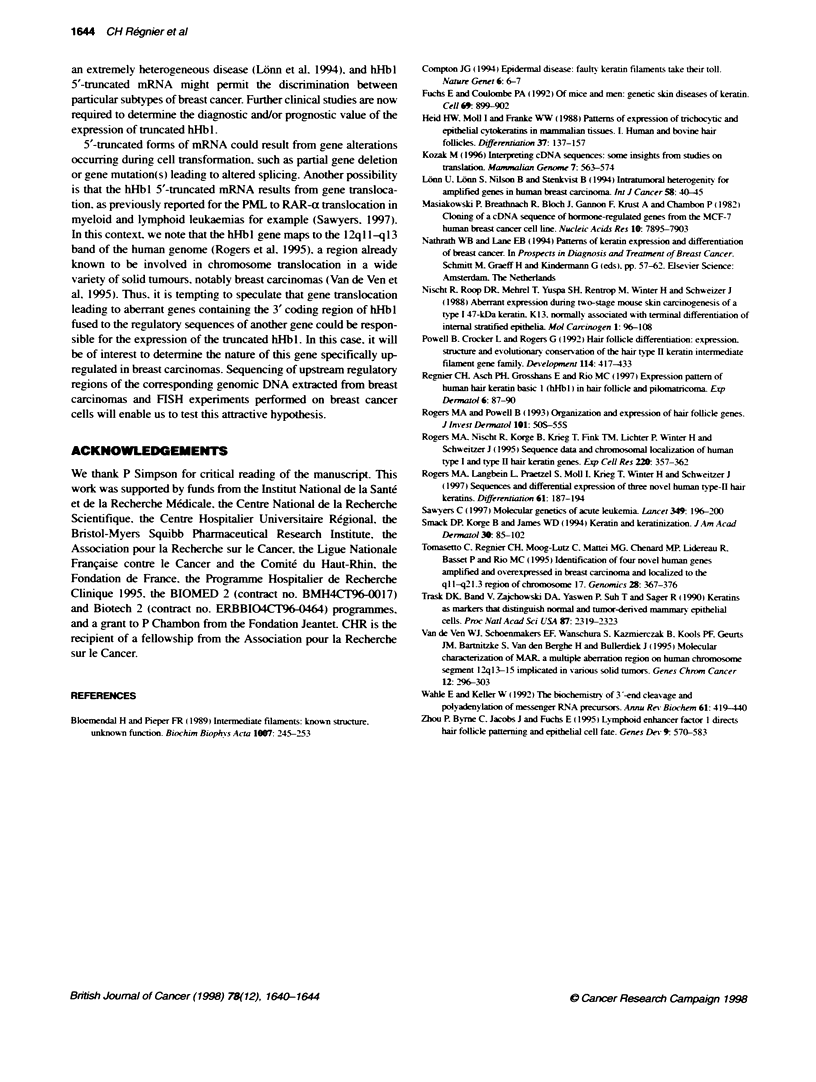# Expression of a truncated form of hHb1 hair keratin in human breast carcinomas.

**DOI:** 10.1038/bjc.1998.736

**Published:** 1998-12

**Authors:** C. H. Régnier, A. Boulay, P. H. Asch, C. Wendling, M. P. Chenard, C. Tomasetto, M. C. Rio

**Affiliations:** Institut de Génétique et de Biologie Moléculaire et Cellulaire, Centre National de la Recherche Scientifique/Institut National de la Santé et de la Recherche Médicale U184/Université Louis Pasteur, Illkirch, France.

## Abstract

**Images:**


British Journal of Cancer (1998) 78(12), 1640–1644

© 1998 Cancer Research Campaign

### Expression of a truncated form of hHb1 hair keratin in human breast carcinomas

**CH Régnier[1], A Boulay[1], PH Asch[2], C Wendling[1], MP Chenard[3], C Tomasetto[1] and MC Rio[1]**

[1]Institut de Génétique et de Biologie Moléculaire et Cellulaire (IGBMC), Centre National de la Recherche Scientifique/Institut National de la Santé et de la Recherche Médicale U184/Université Louis Pasteur, BP 163, 67404 Illkirch Cedex, CU de Strasbourg, France; [2]Clinique Dermatologique des Hôpitaux Universitaires de Strasbourg, 67085 Strasbourg Cedex, France; [3]Service d'Anatomie Pathologique Générale, Centre Hospitalier Universitaire de Hautepierre, 67098 Strasbourg Cedex, France

**Summary** Human hHb1 belongs to the type II hard keratin family and is physiologically expressed in hair shafts. In the present study, using specific 3′ and 5′ probes for hHb1, we established that breast carcinomas ectopically express a hHb1 5′-truncated mRNA, and that this transcript is restricted to malignant epithelial cells. Furthermore, an in vitro study indicated that it could be translated. We concluded that, in breast carcinomas, expression of truncated hHb1 is related to epithelial cell transformation. Because the hHb1 gene maps to 12q11–q13, a chromosome region known to present several breakpoints in solid tumours, we propose that the hHb1 gene might represent a target for such alterations.

**Keywords:** hHb1; hair keratin; breast cancer; in situ hybridization

By differential screening of a cDNA library established from metastatic lymph nodes derived from a human breast cancer, we have previously identified and cloned the MLN 137 cDNA (Tomasetto et al, 1995) (accession no. X80197). This cDNA is 1124 nucleotides long and shows homologies with sheep wool keratin K2-9 (Powell et al, 1992). At the same time, Rogers and co-workers cloned the human hHb1 cDNA counterpart of K2-9 (Rogers et al, 1995). The gene coding for hHb1 has been mapped to the type II keratin cluster at 12q11–q13 (Rogers et al, 1995). Comparison of MLN137 and hHb1 sequences showed that MLN137 corresponds to the 3′ half of the hHb1 cDNA. In situ hybridization to human scalp showed strong hHb1 expression in the cortical cells of the hair shaft, from the lower cortex, slightly above the apex of the dermal papilla, to the upper third of the cortex (Régnier et al, 1997; Rogers et al, 1997). Furthermore, hHb1 expression was also observed in the transitional cell layer in pilomatricoma, a tumour exhibiting follicular differentiation (Régnier et al, 1997).

Keratins belong to a multigene family which contains more than 30 members. They are grouped into type I acidic and type II basic to neutral proteins. The association of equimolar amounts of distinct pairs of type I and type II keratins gives rise to intermediate filaments which are organized in networks in epithelial cells (Bloemendal and Pieper, 1989). Most keratins correspond to soft α-keratins, expressed in various types of epithelia, whereas hard α-keratins are involved in the formation of hair and nails. The hair keratin family is composed of four major type I (Ha1-4) and type II (Hb1-4) keratins, and a minor pair (Hax and Hbx) (Heid et al, 1988). Recently, several other members have been reported for type I and type II hair keratins, showing that these families are more complex than previously assumed (Rogers et al, 1997).

*Received 27 November 1997*

*Revised 28 April 1998*

*Accepted 29 April 1998*

*Correspondence to:* MC Rio, IGBMC, BP 163, Illkirch Cedex, 67404, France

Little is known about the precise mechanisms underlying the regulation of the hair-specific keratins. In addition to recognizing motifs used by many eukaryotic genes in transcription, such as AP1, AP2 and SP1 sites, a number of potential regulatory motifs have been identified. Two of them, referred to as the HK-1 (Rogers and Powell, 1993) and LEF-1 (Zhou et al, 1995) motifs, have been shown to be able to regulate tissue-specific expression of keratins. Furthermore, because keratins form a gene cluster, their expression could, at least partially, be regulated by a locus activation region.

In normal human breast tissue, immunohistochemical analysis showed that the basal and luminal cells of the ducts can be discriminated by the keratins they express. K5 and K14 are specific to myoepithelial cells, whereas luminal cells are characterized by expression of K7, K8, K18 and K19. Several soft keratins are produced in breast carcinomas, and they are used as tumour markers because their profile of expression correlates with different types of epithelial differentiation and function (Trask et al, 1990; Nathrath and Lane, 1994). They can be found intratumourally and in the blood, circulating as partially degraded complexes. High levels of K8, K18 and K19 in breast carcinomas have been correlated with a better prognosis, whereas a decrease of K5 was associated with tumour progression (Trask et al, 1990; Nathrath and Lane, 1994).

Here, we have studied the expression of hHb1 in benign and malignant breast tumours by Northern blot analysis using 3′ and 5′ probes specific for hHb1.

### MATERIALS AND METHODS

### Human cell lines and tissues

Breast cancer cell lines are described and are available in the American Type Culture Collection (ATCC, Rockville, MD, USA). Cells were routinely cultured in Dulbecco's modified Eagle medium supplemented with 10% fetal calf serum.

**Table 1** Clinical and biochemical characteristics of breast tumours expressing MLN137

| Patient | Age | Meno | Grade | ER | PR | pS2 | LN |
|---|---|---|---|---|---|---|---|
| 1 | 26 | – | III | – | – | – | 0/10 |
| 2 | 49 | – | II | – | – | – | 0/9 |
| 3 | 55 | + | II | – | – | – | 0/12 |
| 4 | 32 | – | III | – | – | – | 3/18 |
| 5 | 53 | + | III | + | + | + | 1/12 |
| 6 | 41 | – | III | + | – | – | 2/7 |
| 7 | 70 | + | I | + | + | + | 0/9 |

Meno, menopausal status of the patients; grade, Scarff, Bloom and Richardson tumour grade; ER and PR, oestrogen and progesterone receptors, respectively; –, negative; +, positive; LN, axillary lymph nodes, number of metastatic LN vs number of studied LN.

Surgical specimens were obtained at the Hôpitaux Universitaires de Strasbourg. Depending on subsequent analysis, they were either immediately frozen in liquid nitrogen (RNA extraction), or fixed in phosphate-buffered formalin (4%) and embedded in paraffin (histological analysis and in situ hybridization).

### Construction of a hairy skin cDNA library and cloning of the full-length hHb1 cDNA

Poly$(A^+)$RNA from normal hairy skin was used to construct a cDNA library with the Uni-ZAP XR vector kit (Stratagene, La Jolla, CA, USA). Clones (200 000) were plated and analysed as previously described (Tomasetto et al, 1995). Nitrocellulose filters were hybridized with the MLN137 $^{32}$P-labelled cDNA as a probe. Twenty clones were selected, purified and sequenced. One of them containing 1940 bp corresponded to the complete hHb1 cDNA.

### Preparation of the hHb1 3′ and 5′ specific probes

The hHb1 3′ specific probe was released from MLN137 cDNA by Sau3AI digestion (nt 464–928) and subcloned into pBluescript (Stratagene, La Jolla, CA, USA).

The hHb1 5′ specific probe was obtained by *Eco*RI/*Hin*cII digestion of the full-length hHb1 cDNA (nt 1–327).

### RNA preparation and analysis

RNA preparation and analysis were performed as previously reported (Tomasetto et al, 1995). After extraction using a guanidinium isothiocyanate method, total RNAs were fractionated on 1% agarose gel in the presence of 2.2 M formaldehyde, and transferred to nylon membrane (Hybond N, Amersham Corporation, Arlington Heights, IL, USA). Northern blots were hybridized (5×SSC; 50% formamide; 42°C; 36–48 h) with $^{32}$P-labelled probes. Stringent washings [0.1×SSC; 0.1% sodium dodecyl sulphate (SDS); 60°C] were performed twice. Blots were autoradiographed at –80°C.

**Figure 1** Schematic representation **(A)** and primary sequence **(B)** of the human MLN137 protein. **(A)** The rod domain of hHb1 contains conserved α-helical structures (1A, 1B, 2A and 2B) interrupted by short linker sequences (L1, L12 and L2). It is flanked by the N-terminal head and C-terminal tail domains, specific to hHb1. The relative position of MLN137, 5′ hHb1 and 3′ hHb1 cDNA probes along the full-length hHb1 cDNA are indicated. ORF, open reading frame; UTR, untranslated region. **(B)** cDNA and deduced amino acid sequences of MLN137. Nucleotide residues are numbered from 5′ to 3′ and amino acids in the open reading frame are designated by the one letter code. The underlined nucleotide sequence corresponds to the 3′ hHb1 cDNA probe

*© Cancer Research Campaign 1998* 

**Figure 2** Northern blot analysis of benign and malignant human breast tissues using the hHb1 3′ probe. Each lane contained 10 µg of total RNA. From left to right, RNA samples from SK-BR-3 (lane 1), carcinomas (lanes 2–6), metastases (lanes 7 and 8) and fibroadenomas (lanes 9–11) are loaded. A 1.1-kb transcript is expressed, at various levels, in some carcinomas (lanes 2, 3 and 6), and in one mestastatic sample (lane 7). The 36B4 probe (Masiakowski et al, 1982) was used as positive internal control. Autoradiography was for 2 days for hybridization of 3′ hHb1, whereas 36B4 hybridization was exposed for 16 h. RNA molecular sizes are indicated in kb

**Figure 3** Northern blot analysis of SK-BR-3 human breast cancer cell line, normal human breast skin and scalp, using hHb1 3′ and 5′ probes. 10 µg of total RNA from SK-BR-3 (lane 1), normal breast skin (lane 2) and normal scalp (lane 3) were loaded. Northern blot is successively hybridized with 3′ hHb1 (**A**), 5′ hHb1 (**B**) and 36B4 (**C**) probes. (**A**) 3′ hHb1 probe detected 1.1 kb and 1.9 kb mRNA in SK-BR-3 and scalp respectively. (**B**) 5′ hHb1 probe only detected 1.9 kb mRNA in scalp. No transcripts were detected in breast skin (**A** and **B**). RNA molecular sizes are indicated in kb

### In situ hybridization

In situ hybridization was performed using a $^{35}$S-labelled antisense RNA probe ($5 \times 10^8$ c.p.m. µg$^{-1}$). Formaldehyde-fixed paraffin-embedded tissue sections (6 µm thick) were deparaffinized in LMR (Labo-Moderne, France), rehydrated and digested with proteinase K (1 µg ml$^{-1}$; 30 min, 37°C). Hybridization was for 18 h, followed by RNase treatment (20 µg ml$^{-1}$; 30 min; 37°C) and stringently washed twice ($2 \times$ SSC, 50% formamide; 60°C, 2 h). Autoradiography was for 2–4 weeks using NTB2 emulsion (Kodak). After exposure, the slides were developed and counter-stained using toluidine blue. $^{35}$S-labelled sense transcript was tested in parallel as a negative control.

### In vitro translation

In vitro translation of 1 µg of sense MLN137 RNA was performed using rabbit reticulocyte lysate in the presence of [$^{35}$S]methionine, as described by the manufacturer (Promega, Madison, WI, USA). Sixteen µl of the total volume (50 µl) of the reaction was analysed on 12% sodium dodecyl sulphate polyacrylamide gel electrophoresis (SDS-PAGE). Dried gels were exposed for 16 h at –80°C.

### RESULTS

### Northern blot analysis of benign and malignant breast tumours using a hHb1 3′ probe

Keratin structure can be subdivided into three parts. There is a central rod occurring in a helical conformation, interrupted by three short non-helical linkers whose length and sequence are well conserved in all keratins. This core structure is flanked by amino- (N-) and carboxy- (C-) terminal non-helical regions, the head and tail domains, respectively, which are specific to each keratin (Bloemendal and Pieper, 1989) (Figure 1A). MLN137 cDNA which encodes half of the helical domain, the tail domain and the 3′ untranslated region (UTR) of hHb1 (Figure 1A and B), hybridized with several mRNAs when used as a probe for Northern blot analysis (data not shown). To avoid these cross-reactions, we designed a hHb1 3′ probe, specific to hHb1, containing 464 bp located at the 3′ end of the MLN137 cDNA and corresponding to the tail domain and part of the 3′ UTR.

The hHb1 3′ probe was used to hybridize total RNA extracted from normal and malignant cells and tissues. Among breast cancer cell lines, a unique 1.1-kb mRNA was observed in SK-BR-3, MDA-MB-231 and MCF7, whereas ZR-75-1, BT474, T47D and BT20 showed no hybridization (Figure 2, lane 1, and data not shown). In vivo, 7 out of 34 (20%) primary breast carcinomas expressed the 1.1-kb transcript, to variable intensities (Figure 2, lanes 2, 3 and 6, and data not shown). Clinical and biochemical characteristics including the age and menopausal status of the patients, the presence or absence of lymph node metastases, the tumour hormonodependency as estimated using oestrogen and progesterone receptors and the pS2 marker, and the Scarff, Bloom and Richardson (SBR) tumour grade of the seven positive tumours have been listed in Table 1. No evident relationship could be drawn between MLN 137 overexpression and any of these features of the tumour. One out of the two metastases studied was also positive (Figure 2, lane 7). Breast fibroadenomas (15 cases studied) as well as normal adult lung, stomach, colon, liver, kidney and placenta were negative (Figure 2, lanes 9–11, and data not shown).

On Northern blots, the transcript detected using the hHb1 3′ probe exhibited a size of 1.1 kb. This size was not in accordance with that of about 2 kb deduced from the full-length hHb1 cDNA (Rogers et al, 1995). However, it fits with that of the MLN137 cDNA (1124 bp) cloned from a breast cancer metastasis, suggesting that breast carcinomas express a 5′-truncated form of hHb1 mRNA.

### Cloning of the hHb1 5′ probe

To test this hypothesis, we cloned the full-length hHb1 cDNA and designed a hHb1 5′ specific probe. Using in situ hybridization, we had previously observed that the hHb1 3′ riboprobe hybridized to a transcript present in hair shafts (Régnier et al, 1997). We assumed that, at this physiological site of expression, the transcript detected should correspond to the complete hHb1 mRNA. Thus, we constructed a scalp cDNA library (see Materials and methods). Its screening, using the MLN137 probe, led to the identification and cloning of a full-length hHb1 cDNA. A 327-bp fragment of the 5′ end of this cDNA corresponding to the 5′ UTR and part of the head of hHb1 was subcloned to produce a hHb1 5′ specific probe (Figure 1A).

 © Cancer Research Campaign 1998

**Figure 4** In situ hybridization of normal and malignant breast tissues using a hHb1 3′ riboprobe. Sections of normal breast (**A**) and invasive tumour (**B**) were hybridized with antisense $[^{35}S]$RNA probe specific for 3′ hHb1. (**A**) Normal ducts are devoid of signal. (**B**) The probe detected a transcript strongly expressed in the tumoral epithelial cells, whereas the stromal part of the tumour was totally negative. No significant labelling above background was found when using sense human 3′ hHb1 RNA probe (data not shown). Bright field (**A** and **B**)

**Figure 5** SDS-PAGE of in vitro translation of MLN 137 RNA. 16 μl of in vitro translation reaction using sense (lane 1) and antisense (lane 2) MLN 137 RNA are loaded. A 27-kDa protein is produced using sense RNA (lane 1)

### Breast carcinomas expressed a hHb1 5′-truncated mRNA

3′ and 5′ hHb1 probes were used for Northern blot analysis of RNA samples extracted from the SK-BR-3 breast cancer cell line, and from normal breast skin and scalp (Figure 3). Whereas the hHb1 3′ probe hybridized to transcripts of 1.1 kb and 1.9 kb in SK-BR-3 and scalp, respectively, the hHb1 5′ probe only detected the 1.9-kb mRNA in scalp. As expected, normal breast skin was devoid of the 1.1-kb and 1.9-kb transcripts. Moreover, using the hHb1 5′ probe, none of the normal and malignant tissue samples previously tested with the hHb1 3′ probe (Figure 2) gave positive signals. Together, these findings showed that the 1.1-kb mRNA, detected in breast carcinomas with the hHb1 3′ probe, but not with the hHb1 5′ probe, corresponds to a 5′-truncated form of the 1.9-kb hHb1 mRNA normally expressed in hairy skin.

Using a hHb1 3′ riboprobe, in situ hybridization showed that the hHb1 5′-truncated mRNA was specifically expressed by malignant epithelial cells in both primary and secondary tumours (Figure 4). The stromal compartment of the tumours as well as normal ducts were negative. These results indicate that the ectopic expression of hHb1 5′-truncated mRNA is related to the malignant status of mammary epithelial cells.

### In vitro translation of the hHb1 5′-truncated (MLN137) mRNA

Analysis of its sequence showed that MLN137 cDNA encodes an open reading frame. A classical AATAAA poly(A) addition signal sequence (Wahle and Keller, 1992) was located 11 bp upstream of the poly(A) stretch (Figure 1B). Moreover, two potential sites of initiation of translation were present, giving rise to putative proteins corresponding to either the last 202 or 235 amino acids of the C-terminal part of the hHb1 keratin. To determine whether these sites are functional, we performed in vitro translation of the MLN137 mRNA. Sense MLN137 RNA gave rise to a band of 27 kDa, whereas no band was observed using MLN137 antisense RNA as control (Figure 5). A protein size of 27 kDa is consistent with a 235-amino acid long protein. Accordingly, the first ATG codon had the most favourable context for initiation of translation (Kozak, 1996). Thus, the MLN137 mRNA corresponding to the 5′-truncated form of hHb1 mRNA is translated in vitro, suggesting that a N-truncated hHb1 keratin could be synthesized in vivo in some breast carcinomas.

### DISCUSSION

From a breast cancer metastasis cDNA library, we have previously cloned the MLN137 cDNA (Tomasetto et al, 1995) corresponding to the 3′ end of the hHb1 hair keratin cDNA (Rogers et al, 1995). In the present study, using two probes specific to the 5′ and 3′ ends of the hHb1 cDNA, we established that breast carcinomas specifically express a 1.1-kb 5′-truncated form of the hHb1 mRNA. In addition, an in vitro translation study showed that a N-truncated hHb1 protein can be translated. The hHb1 5′-truncated mRNA was not present in the hair shaft which expresses the wild-type 1.9-kb hHb1 mRNA. With the exception of hairy skin, all normal tissues tested so far were devoid of 5′-truncated or wild-type hHb1 mRNA. Moreover, in breast carcinomas, the hHb1 5′-truncated transcript is restricted to malignant epithelial cells, showing that its expression is related to epithelial cell transformation.

The physiological function of keratins is the distribution of mechanical forces between cells and tissues, and ultimately the maintenance of epithelial cell integrity. Human soft and hard keratins are specific to epithelia and hairs and nails respectively. Intermediate filaments result from the stoichiometric assemblage of specific type I and type II keratins (Bloemendal and Pieper, 1989; Rogers et al, 1997). Abberent forms of soft keratin have been shown to be responsible for several epidermal genetic diseases, notably through the formation of dominant negative heterodimers (Fuchs and Coulombe, 1992; Compton, 1994). In breast carcinomas, profiles of soft keratin expression are used as tumour markers to discriminate various subtypes of tumours, because they correlate with different statuses of epithelial cell differentiation (Trask et al, 1990; Nathrath and Lane, 1994). Finally, aberrant expression of the soft keratin K13, normally associated with the terminal differentiation of internal stratified epithelia, has been reported in mouse skin tumours (Nischt et al, 1988).

Very little is known concerning pathological expression of hard keratins (Smack et al, 1994). Using in situ hybridization, the hHb1 3′ probe revealed the presence of a transcript in pilomatricoma indicating that they are tumours of the hair follicle differentiation type (Régnier et al, 1997). However, further studies are needed to determine whether this transcript corresponds to the wild-type or the 5′-truncated form of hHb1. In breast carcinomas, this is the first report of an ectopic expression of hard keratin-related gene. About 20% of invasive breast carcinomas and some of the derived metastases expressed the hHb1 5′-truncated mRNA. No evident association between hHb1 5′-truncated mRNA and classical clinical or biochemical tumour parameters can be drawn. Breast carcinoma is

© Cancer Research Campaign 1998 

an extremely heterogeneous disease (Lönn et al. 1994), and hHb1 5′-truncated mRNA might permit the discrimination between particular subtypes of breast cancer. Further clinical studies are now required to determine the diagnostic and/or prognostic value of the expression of truncated hHb1.

5′-truncated forms of mRNA could result from gene alterations occurring during cell transformation, such as partial gene deletion or gene mutation(s) leading to altered splicing. Another possibility is that the hHb1 5′-truncated mRNA results from gene translocation, as previously reported for the PML to RAR-α translocation in myeloid and lymphoid leukaemias for example (Sawyers, 1997). In this context, we note that the hHb1 gene maps to the 12q11–q13 band of the human genome (Rogers et al, 1995), a region already known to be involved in chromosome translocation in a wide variety of solid tumours, notably breast carcinomas (Van de Ven et al, 1995). Thus, it is tempting to speculate that gene translocation leading to aberrant genes containing the 3′ coding region of hHb1 fused to the regulatory sequences of another gene could be responsible for the expression of the truncated hHb1. In this case, it will be of interest to determine the nature of this gene specifically up-regulated in breast carcinomas. Sequencing of upstream regulatory regions of the corresponding genomic DNA extracted from breast carcinomas and FISH experiments performed on breast cancer cells will enable us to test this attractive hypothesis.

### ACKNOWLEDGEMENTS

We thank P Simpson for critical reading of the manuscript. This work was supported by funds from the Institut National de la Santé et de la Recherche Médicale, the Centre National de la Recherche Scientifique, the Centre Hospitalier Universitaire Régional, the Bristol-Myers Squibb Pharmaceutical Research Institute, the Association pour la Recherche sur le Cancer, the Ligue Nationale Française contre le Cancer and the Comité du Haut-Rhin, the Fondation de France, the Programme Hospitalier de Recherche Clinique 1995, the BIOMED 2 (contract no. BMH4CT96-0017) and Biotech 2 (contract no. ERBBIO4CT96-0464) programmes, and a grant to P Chambon from the Fondation Jeantet. CHR is the recipient of a fellowship from the Association pour la Recherche sur le Cancer.

### REFERENCES

Bloemendal H and Pieper FR (1989) Intermediate filaments: known structure, unknown function. *Biochim Biophys Acta* **1007**: 245–253

Compton JG (1994) Epidermal disease: faulty keratin filaments take their toll. *Nature Genet* **6**: 6–7

Fuchs E and Coulombe PA (1992) Of mice and men: genetic skin diseases of keratin. *Cell* **69**: 899–902

Heid HW, Moll I and Franke WW (1988) Patterns of expression of trichocytic and epithelial cytokeratins in mammalian tissues. I. Human and bovine hair follicles. *Differentiation* **37**: 137–157

Kozak M (1996) Interpreting cDNA sequences: some insights from studies on translation. *Mammalian Genome* **7**: 563–574

Lönn U, Lönn S, Nilson B and Stenkvist B (1994) Intratumoral heterogenity for amplified genes in human breast carcinoma. *Int J Cancer* **58**: 40–45

Masiakowski P, Breathnach R, Bloch J, Gannon F, Krust A and Chambon P (1982) Cloning of a cDNA sequence of hormone-regulated genes from the MCF-7 human breast cancer cell line. *Nucleic Acids Res* **10**: 7895–7903

Nathrath WB and Lane EB (1994) Patterns of keratin expression and differentiation of breast cancer. In *Prospects in Diagnosis and Treatment of Breast Cancer*, Schmitt M, Graeff H and Kindermann G (eds), pp. 57–62. Elsevier Science: Amsterdam, The Netherlands

Nischt R, Roop DR, Mehrel T, Yuspa SH, Rentrop M, Winter H and Schweizer J (1988) Aberrant expression during two-stage mouse skin carcinogenesis of a type I 47-kDa keratin, K13, normally associated with terminal differentiation of internal stratified epithelia. *Mol Carcinogen* **1**: 96–108

Powell B, Crocker L and Rogers G (1992) Hair follicle differentiation: expression, structure and evolutionary conservation of the hair type II keratin intermediate filament gene family. *Development* **114**: 417–433

Regnier CH, Asch PH, Grosshans E and Rio MC (1997) Expression pattern of human hair keratin basic 1 (hHb1) in hair follicle and pilomatricoma. *Exp Dermatol* **6**: 87–90

Rogers MA and Powell B (1993) Organization and expression of hair follicle genes. *J Invest Dermatol* **101**: 50S–55S

Rogers MA, Nischt R, Korge B, Krieg T, Fink TM, Lichter P, Winter H and Schweitzer J (1995) Sequence data and chromosomal localization of human type I and type II hair keratin genes. *Exp Cell Res* **220**: 357–362

Rogers MA, Langbein L, Praetzel S, Moll I, Krieg T, Winter H and Schweitzer J (1997) Sequences and differential expression of three novel human type-II hair keratins. *Differentiation* **61**: 187–194

Sawyers C (1997) Molecular genetics of acute leukemia. *Lancet* **349**: 196–200

Smack DP, Korge B and James WD (1994) Keratin and keratinization. *J Am Acad Dermatol* **30**: 85–102

Tomasetto C, Regnier CH, Moog-Lutz C, Mattei MG, Chenard MP, Lidereau R, Basset P and Rio MC (1995) Identification of four novel human genes amplified and overexpressed in breast carcinoma and localized to the q11–q21.3 region of chromosome 17. *Genomics* **28**: 367–376

Trask DK, Band V, Zajchowski DA, Yaswen P, Suh T and Sager R (1990) Keratins as markers that distinguish normal and tumor-derived mammary epithelial cells. *Proc Natl Acad Sci USA* **87**: 2319–2323

Van de Ven WJ, Schoenmakers EF, Wanschura S, Kazmierczak B, Kools PF, Geurts JM, Bartnitzke S, Van den Berghe H and Bullerdiek J (1995) Molecular characterization of MAR, a multiple aberration region on human chromosome segment 12q13–15 implicated in various solid tumors. *Genes Chrom Cancer* **12**: 296–303

Wahle E and Keller W (1992) The biochemistry of 3′-end cleavage and polyadenylation of messenger RNA precursors. *Annu Rev Biochem* **61**: 419–440

Zhou P, Byrne C, Jacobs J and Fuchs E (1995) Lymphoid enhancer factor 1 directs hair follicle patterning and epithelial cell fate. *Genes Dev* **9**: 570–583

 © Cancer Research Campaign 1998